# Structure and dynamics of enterovirus genotype networks

**DOI:** 10.1126/sciadv.ado1693

**Published:** 2024-06-19

**Authors:** Nathânia Dábilla, Patrick T. Dolan

**Affiliations:** Quantitative Virology and Evolution Unit, Laboratory of Viral Diseases, NIAID, NIH, Bethesda, MD, USA.

## Abstract

Like all biological populations, viral populations exist as networks of genotypes connected through mutation. Mapping the topology of these networks and quantifying population dynamics across them is crucial to understanding how populations adapt to changes in their selective environment. The influence of mutational networks is especially profound in viral populations that rapidly explore their mutational neighborhoods via high mutation rates. Using a single-cell sequencing method, scRNA-seq–enabled acquisition of mRNA and consensus haplotypes linking individual genotypes and host transcriptomes (SEARCHLIGHT), we captured and assembled viral haplotypes from hundreds of individual infected cells, revealing the complexity of viral population structures. We obtained these genotypes in parallel with host cell transcriptome information, enabling us to link host cell transcriptional phenotypes to the genetic structures underlying virus adaptation. Our examination of these structures reveals the common evolutionary dynamics of enterovirus populations and illustrates how viral populations reach through mutational “tunnels” to span evolutionary landscapes and maintain connection with multiple adaptive genotypes simultaneously.

## Teaser

SEARCHLIGHT enables observation of virus population structure and dynamics, revealing alternative evolutionary paths.

## INTRODUCTION

Single-cell RNA sequencing (scRNA-seq) methods enable monitoring of transcriptional dynamics (*1*, *2*), virus infection (*3*), and the heterogeneity of viral and host cell populations (*1*, *4*–*7*). In vitro, combined capture of viral and host transcripts with scRNA-seq has allowed us to correlate virus transcript abundance with host responses (*4*). In vivo, these methods have identified host cell tropism (*8*) and molecular signatures associated with severe disease (*3*).

Because of limitations in the processivity of the reverse transcriptase used in library generation, studies of viral genotypes using scRNA-seq platforms have largely been limited to influenza A virus (IAV), with a genome consisting of small segments (up to 2300 bases in length) (*9*) and amenable to amplification directly from cDNA pools or fragments of viral genomes (*10*). Characterization of heterogeneity in IAV populations has demonstrated that stochastic fluctuations in segment copy number influence innate immune responses (*5*, *11*). Attempts to capture viral genotypes from longer viral genomes have been limited (*6*, *10*). In all cases, viral population structure and mutational spectra have not been thoroughly explored. Capturing viral genotypes would unlock investigations of virus population structure and dynamics, notably, in ways that connect viral genotype with host cell type and transcriptional state.

To adapt existing single-cell pipelines to the generalized capture of viral genotype information, we developed scRNA-seq–enabled acquisition of mRNA and consensus haplotypes linking individual genotypes and host transcriptomes (SEARCHLIGHT), wherein custom, virus-specific reverse transcription (RT) primers tiled at regular intervals across the viral genome are used to generate cDNA transcripts. Including these primers during microfluidic droplet generation enables the reconstruction of the consensus viral genotypes present within each cell. Combining this modification with high-accuracy long-read sequencing, we reconstruct viral genotypes from hundreds of individual infected cells in parallel to matched host cell transcriptomes.

Here, we examine the structure of enterovirus (EV) populations derived from long-term passage and an in vitro–transcribed RNA molecular clone, demonstrating the marked difference between the genetic structures of the populations from these two origins. We then examine the dynamics of adapting EV-A71 populations during in vitro passage to understand how experimental populations explore the genotypic network. Even in this simple model of viral infection and evolution, SEARCHLIGHT reveals complex linkage interactions between low-frequency variants in the viral population and highlights the multiple paths the virus explores while adapting to selective pressures.

## RESULTS

### Capturing viral genotypes from individual infected cells

**Fig. 1.**
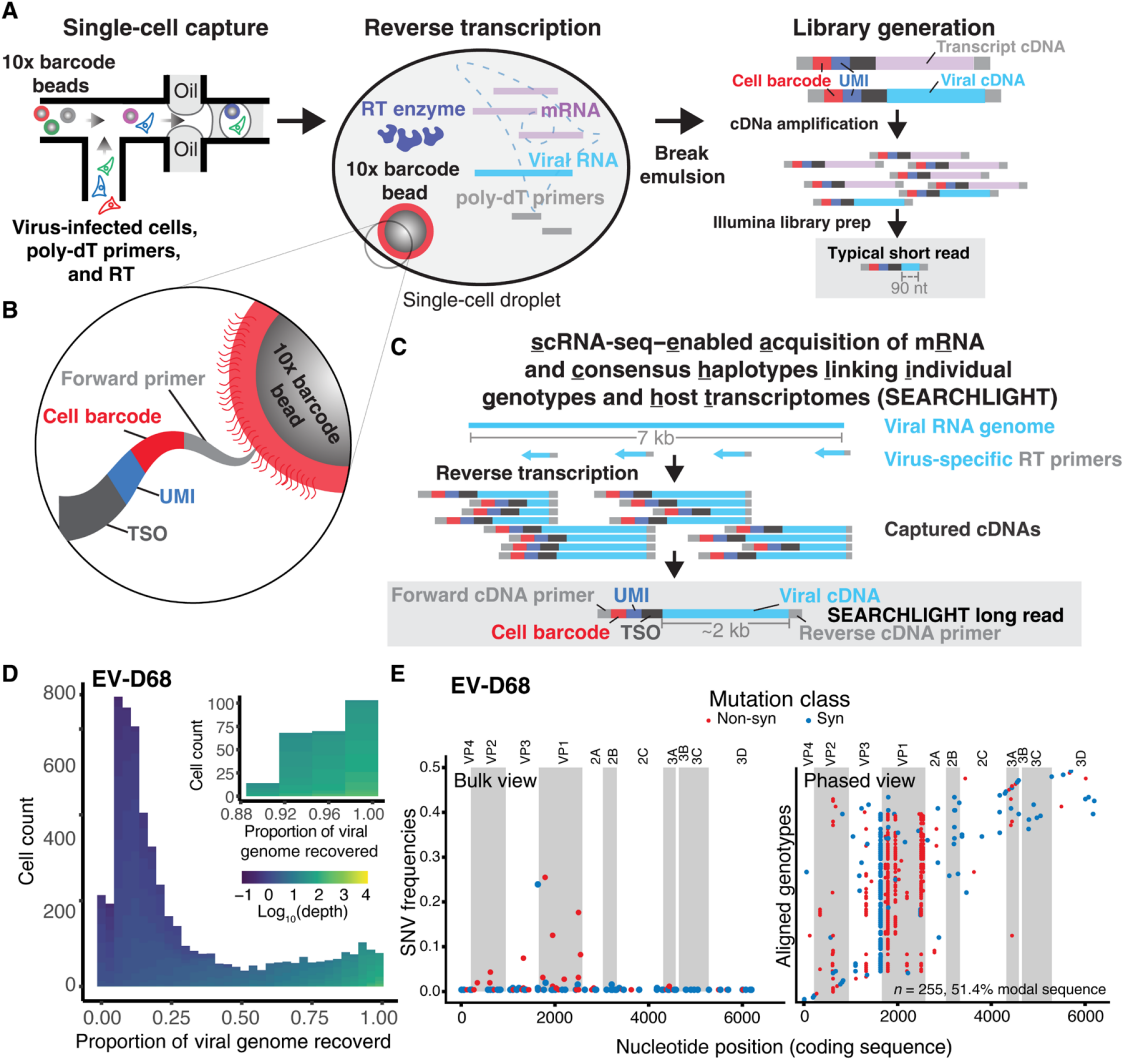
Traditional single-cell sequencing and the development of SEARCHLIGHT. (**A**) Conventional method: cDNAs generated in each droplet are processed into short-read libraries, typically capturing 90 to 200 nucleotides (nt) of viral sequence in each read, primarily from the 3′ end of a polyadenylated transcript. (**B**) The gel bead captured along with individual cells barcodes newly synthesized cDNA via the template-switch sequence encoded in the oligonucleotides conjugated to each bead, marking the cDNA with the cell barcode and unique molecular identifier (UMI). (**C**) SEARCHLIGHT method: Barcoded cDNAs from tiled, virus-specific primers are used to generate long-read sequencing libraries that cover the entire viral genome, yielding ~2 kb of viral sequence per read. (**D**) Histogram of the proportion of viral genome captured in each cell during SEARCHLIGHT sequencing of EV-D68–infected RD cells. Fill color represents the average depth of coverage across the viral genome in each cell. The inset shows the cells from which consensus haplotypes were recovered for subsequent analysis. (**E**) The bulk view of population diversity (left) and the phased haplotype alignment (right). In the haplotype alignment, each haplotype derived from an individual cell is shown as a single row, with differences from the modal genotype shown as points. Color indicates non-synonymous or synonymous mutations. Viral proteins are labeled and denoted by alternate shading.

Microfluidic single-cell sequencing methods partition cells into droplets for RT of mRNA, usually by primers that bind transcript 3′ polyadenylate tails. These primers also capture polyadenylated viral genomes and transcripts, but sequencing library generation and limitations in read length result in small fragments of the viral sequence being captured in downstream short-read sequencing (Fig. 1A). This is sufficient to identify cells with viral RNA content and monitor the transcriptional state of virus-infected and bystander cell populations but not to recover the viral genotype from infected cells.

The template-switch oligonucleotide (TSO) conjugated to the Gel Beads in the 10x Genomics 5′ scRNA-seq protocol (Fig. 1B) captures and barcodes transcripts generated during cDNA synthesis, addressing each read to an individual source cell and transcript by a “cell barcode” and “unique molecular identifier” (UMI), respectively. We add virus-specific primers tiled at regular 2-kb intervals across the viral RNA genome alongside poly-dT primers during in-droplet RT. Taking advantage of the TSO, RT generates barcoded cDNA transcripts (of ~2 kb) covering the entire viral genome, which are subsequently amplified for long-read sequencing. Short-read and long-read library preparation from the same barcoded scRNA-seq cDNA pools allows us to reconstruct the consensus genotype from each cell (Fig. 1C) and map them directly onto the transcriptome and viral RNA count information from matched infected cells (fig. S1).

From a single-cell experiment capturing 8000 cells, with ~30% (2400) infected cells, we recover nearly full-length sequences from about 300 to 400 cells per sample (Fig. 1D and fig. S2) or roughly 10 to 15% of the infected cells. For each sample, we limited our analysis to cells from which we recovered the full coding sequence of each virus genome at sufficient depth per site (10-fold) (Fig. 1D and fig. S2). The consensus viral sequence in each cell was determined on the basis of coverage varying from 10- to 1000-fold with a mean Q score above 50 (10^−5^ errors per base) and a median greater than 42. Revealing linkage relationships across the whole sequence, SEARCHLIGHT transforms our view of the viral population from a conventional “bulk” view of viral populations, where individual alleles are viewed as independent low-frequency variants, to a fully “phased” view, where each cell provides one sequence in a sequence alignment (Fig. 1E).

### Articulating the structure of viral populations

**Fig. 2.**
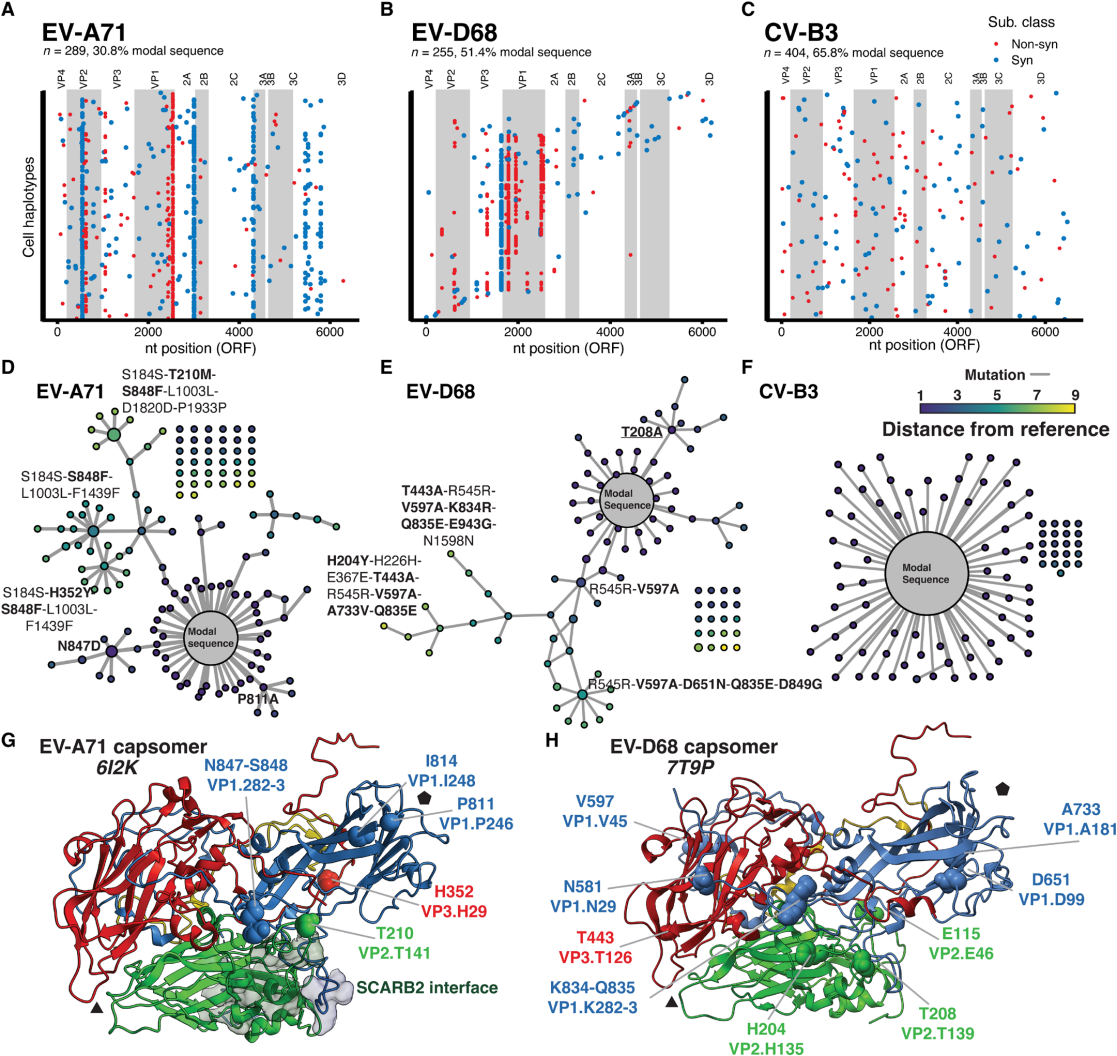
Population dynamics across the EV-A71 genotype network. (**A** to **C**) SEARCHLIGHT-derived haplotypes of three populations of EV-A71, EV-D68, and CV-B3. Color indicates whether mutations were non-synonymous or synonymous. Viral gene products within the viral polyprotein are labeled and denoted by alternate shading. ORF, open reading frame. (**D** to **F**) The same SEARCHLIGHT-derived genotypes, shown as genotypic networks. Each node represents a genotype, with size representing the relative frequency of the genotype. Edges represent single-nucleotide substitutions linking individual genotypes. The unconnected dots are detected genotypes within the sample that are not directly connected through mutation to the modal sequence via other observed genotypes. While the EV-A71 and EV-D68 populations are derived from stocks generated by long-term propagation, the CV-B3 population is derived from a molecular clone. (**G** and **H**) Structures of a single homooligomeric monomer of the (G) EV-A71 and (H) EV-D68 capsid, known as a capsomer. The most frequent mutations found on the recovered haplotypes are highlighted on the structure and colored by subunit. The black triangle and pentagon indicate the three- and fivefold axes, respectively.

We experimentally validated our approach by comparing the sequence and structure of enterovirus populations from distinct origins, either from long-term passage (from the repository stocks) or from in vitro–transcribed RNA from plasmids encoding full-length viral cDNA. We optimized primer sets to perform SEARCHLIGHT on three EV strains: EV-A71 (strain Tainan/4643), Coxsackievirus B3 (strain Nancy), and EV-D68 (strain MO/14-18947), belonging to three distinct EV species (A, B, and D) (table S1). We infected cultured, muscle-derived rhabdomyosarcoma (RD) cells with each viral stock at a multiplicity of infection (MOI) of 0.3 (~2.4 × 10^6^ cells with 8 × 10^5^ infectious units of virus [determined by 50% tissue culture infectious dose (TCID_50_) assay]. After microfluidic single-cell capture targeting 8000 cells, library generation, and sequencing, we recovered consensus genotypes from 289, 255, and 404 individual cells infected with EV-A71, EV-D68, and CV-B3, respectively. We used these data to construct alignments for each virus population (Fig. 2, A to C, and fig. S3).

A large proportion of the cells carried the modal genotype, the most common genotype in the population. In quasi-species literature, this most common genotype is often referred to as the master genotype (*12*–*14*). We propose modal genotype as a more accurate and inclusive term. This genotype is distinct from the consensus sequence that represents an aggregate of the most common nucleotides at each position. The movement of the consensus relative to the modal genotype is a key feature of viral quasi-species; the consensus sequence may not change, although the underlying genotypes may be highly dynamic. In some circumstances, the “consensus genotype” may not exist but is an aggregate of cocirculating genotypes.

The frequency of the modal sequence is variable between samples (ranging from 30.8 and 53.0% for EV-A71 and EV-D68, respectively, to 65.8% for the CV-B3 population). This larger proportion of the modal genotype in the CV-B3 population and the number of single, unlinked single-nucleotide variations (SNVs) reflects its origins, being derived from a molecular clone plasmid. This population is taking its mutational “first steps” in the selective landscape. The EV-A71 and EV-D68 populations showed greater diversity, with more mutations per genome relative to the modal genotype. Notably, bulk RNA-seq would reveal little diversity in any of our sequenced populations, based on the common limit of detection of 1 to 10% in frequency determined by the error rate associated with library preparation. However, the partitioning of individual infected cells, followed by high-depth, high-accuracy sequencing of the barcoded cDNA, allows us to identify genotypes at lower frequencies, even when present as the consensus genotype in only a single cell. This establishes a limit of detection that is determined, instead, by the number of cells collected in the assay and provides a comprehensive census of the genotype diversity and structural complexity of the populations.

Observing single-cell haplotypes in each of these populations enables us to construct a genotypic network describing the mutations (edges) linking individual genotypes (nodes) within the populations (Fig. 2, D to F). This representation of the population as a network highlights the structure of subpopulations connected by SNVs to the modal genotype (Fig. 2, D to F). EV-A71 and EV-D68 populations show much more complex network topologies compared to the CV-B3 population, consistent with the origins of these isolates from long-term passaged repository stocks. Long-term passage has allowed the viral populations to diversify along multiple mutational paths, spanning up to 9 mutations away from the modal sequence (shown in gray). In contrast, the CV-B3 population exhibits a simple hub-and-spoke topology with many genotypes surrounding the modal sequence linked by single mutations.

This result demonstrates the substantial differences between long-term repository-derived populations and those generated from infectious molecular clones. It also highlights the potential hazard associated with using diverse stock populations, especially if one is deriving clonal isolates from stock populations, where each purified isolate may differ significantly from the sequence consensus obtained through next-generation sequencing.

### Optimizing cell engagement through multiple distinct mutational paths

The topology of the EV-A71 genotypic network immediately reveals multiple mutational paths that link substitutions together. Although minor non-synonymous variants T210M (VP2.141), N847D (VP1.282), and S848F (VP1.283) would be apparent from bulk sequencing of this stock population (fig. S3A), the single-cell haplotypes recovered by SEARCHLIGHT reveal a complex linkage relationship between these and other minor variants in the population (Fig. 2A). Placing these mutations on the structure of the EV-A71 capsomer reveals that these sites—210, 847, and 848—although distant in sequence, lie close to one another on the viral capsid surface, at the perimeter the binding interface with the viral receptor, scavenger receptor B2 (*15*). This might suggest that these mutations tune host cell engagement or capsid uncoating kinetics (Fig. 2G). Two other non-synonymous substitutions accumulate separately, P811A (VP1.246) and I814V (VP1.249), both located near the fivefold axis.

### Finding common constraints among EVs

The non-synonymous diversity in EV-D68 was similarly focused on the capsid proteins in the P1 region (Fig. 2, B and H). Notably, several of the mutations in the capsid were similar in location to those identified in the EVA-71 population (Fig. 2, underlined labels). These included H204Y and T208A in VP2 and K834R and Q835E in VP1. Given that these viruses use distinct repertoires of cellular attachment factors and receptors, it was somewhat unexpected to see parallel evolutionary patterns in the capsid. However, mapping these substitutions on the structure of the EV-D68 capsid (*16*) revealed that they either directly overlap with (T208A, VP2.T139) or are located nearby to escape variants identified in a study of neutralizing antigenic sites (K834R and Q835E, near the antigenic site at VP1.285). The T208 site in VP2 represents a shared antigenic site between the EV-D68 capsid and those of EV-A71 (at T210) and CV-A10 (*17*).

### Monitoring the emergence of a new modal genotype in EV-A71

**Fig. 3.**
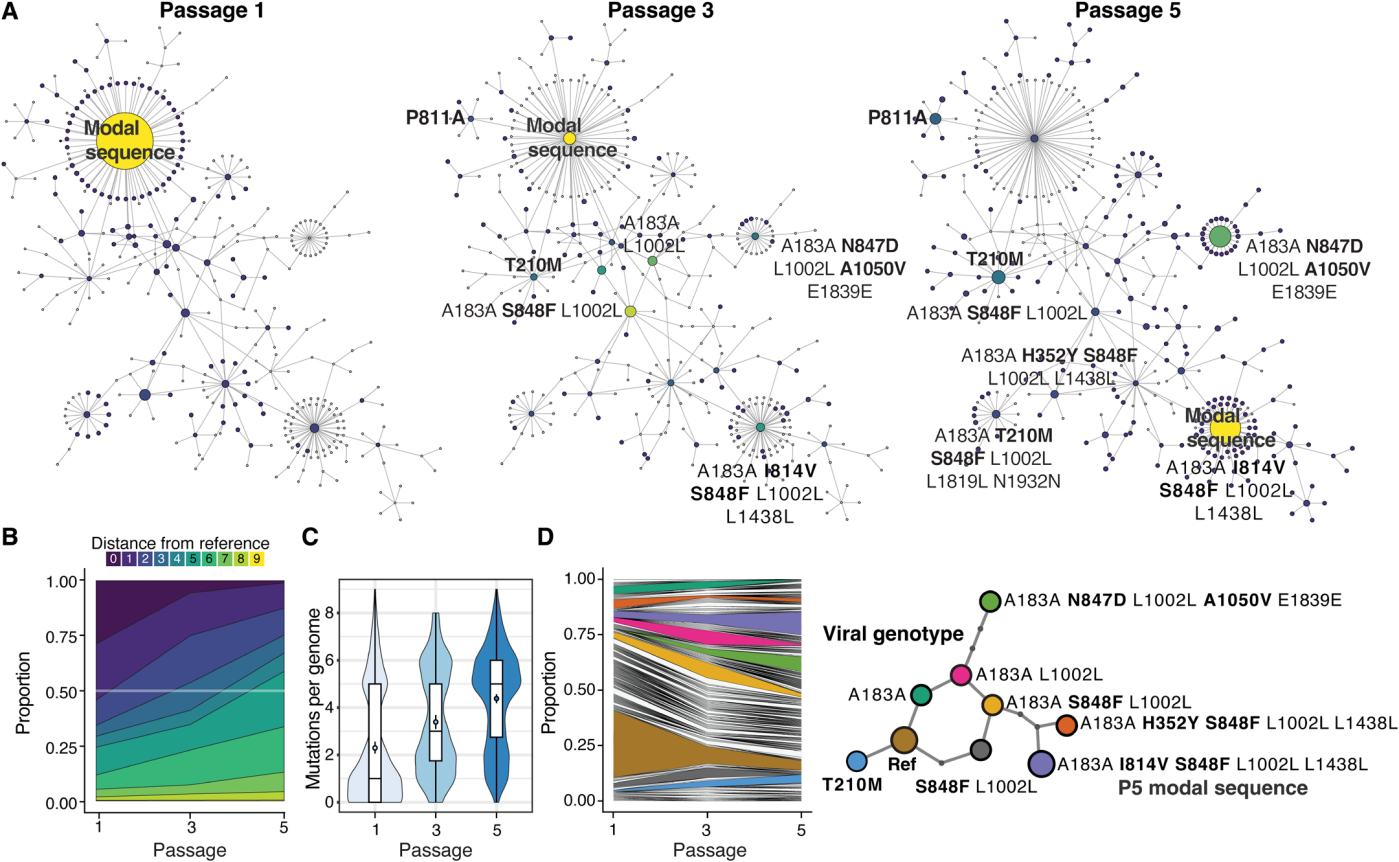
Population dynamics across the EV-A71 genotype network. (**A**) The distribution of genotypes across the mutational network of EV-A71 across passages. The color indicates the relative frequency of genotypes within the population at each passage. The size represents the number of cells infected with that genotype. The “modal genotype,” or the most common genotype, is labeled. By P5, the modal sequence has shifted five mutations from the original modal sequence. (**B**) Plot showing the genetic distance of genotypes in the population relative to the modal sequence. (**C**) Violin plot and boxplot showing the distribution, mean, and median mutations per genome relative to the P1 modal genotype. Point indicates mean mutations per genome, and box shows the median and interquartile range of the population. (**D**) A genotype frequency plot showing the distribution and dynamics of specific genotypes in the population over passage. Major genotypes in the network are highlighted, and their relationship is shown in the subway map representation of the major genotypes in the network.

To understand how the evolutionary dynamics play out across genotype networks in a simple model of evolution, we performed in vitro passage of the EV-A71 population in RD cells. Using virus stocks produced from passage 1, passage 3, and passage 5, we conducted a SEARCHLIGHT experiment targeting 5000 cells each with ~30% infected (MOI = 0.3). We captured nearly full-length sequences from between 220 and 500 cells (fig. S2). The coverage of the viral genome in each cell varied from 10× to 1000×, with *Q* score of mean above 40 (median > 35). From that, we could trace the fate of hundreds of genotypes (Fig. 3).

In five passages, the hierarchy of genotypes in the population completely shifts, giving rise to an alternative modal genotype many mutational steps away from the initial modal genotype (Fig. 3, A to D). This genotype encodes two non-synonymous mutations: S848F (VP1.283), a dominant allele in the original population, and I814V (VP1.248). The frequency of the initial modal genotype from the first passage declines throughout the passages, from nearly 30% to just 1% (fig. S3). Two other genotypes also emerge to high relative frequencies, one combining capsid substitutions S848F (VP1.283) and H352Y (VP3.29) and another carrying a substitution at nearby N847D (VP1.282), with a second in 2B (A1050V). Mutated sites are highlighted on the structures in Fig. 2.

Notably, we observed mean mutations per genome of approximately two (median of 1) (Fig. 3C), which correlates with previous studies conducted in EVs (*18*–*20*), corroborating our findings. The population accumulates approximately 1 mutation per genome per passage relative to the P1 modal genotype, despite no genotypes sweeping through the populations over the course of passage.

### Linking the transcriptional dynamics of infected cells with viral genotype

**Fig. 4.**
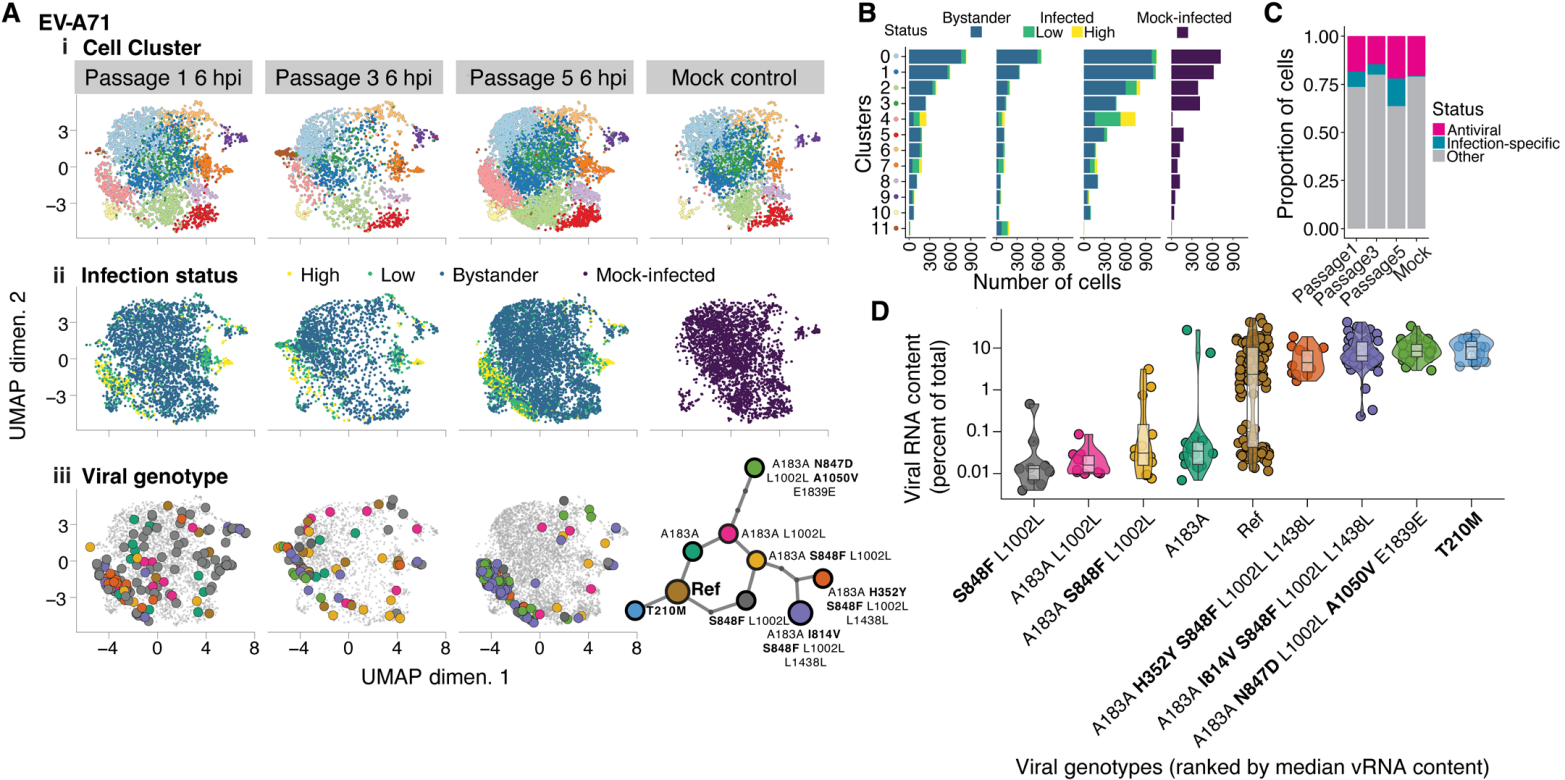
Connecting genotype with infected cell phenotype. (**A**) Uniform manifold approximation projection (UMAP) of the cell transcriptional phenotypes recovered from the passaged cell populations. (i) Clustering of transcriptional phenotypes derived from captured cells. (ii) Identification of infected and uninfected cells. Cells are also classified by high and low viral RNA content. (iii) Major genotypes captured by SEARCHLIGHT placed onto the phenotypic map of the host cells. (**B**) Distribution of transcriptional cluster membership and the proportion of cells in each cluster with no, low, or high viral RNA content. (**C**) Comparison of the proportion of cells either in an antiviral or infection-specific cluster. (**D**) Viral RNA (vRNA) content associated with cells infected with each of the nine major genotypes identified in the passage experiment. Genotypes are ranked by median viral RNA content. Non-synonymous substitutions in each genotype are highlighted with bold lettering.

In parallel to the viral genotypes captured by SEARCHLIGHT, we capture cell transcriptomic phenotypes through conventional single-cell sequencing, allowing us to examine adaptation in the context of host cell heterogeneity. We first clustered cells by their transcriptional states to identify cell phenotypes associated with infection and immune responses, identifying characteristic marker genes for cells in each cluster. Consistent with other studies using infected cultured cells (*4*, *5*, *8*, *11*, *21*), we identified marked diversity in cellular transcriptomes, reflecting subpopulations sampling distinct states (Fig. 4A i and fig. S4). Cells were also classified as infected (low or high viral RNA) or bystanders (Fig. 4, Aii and B, and fig. S4), based on the number of viral reads relative to the background.

On the basis of these assignments, clusters 2 and 5 were associated with innate immune and antiviral markers (table S2), including interferon-stimulated gene 15 (*ISG15*), *ISG20*, and *Chemokine* (*C-C motif*) *ligand *5 (*CCL5*). Cluster 2 expressed a larger repertoire of ISGs, with a profile including *Interferon-Induced Protein With Tetratricopeptide Repeats 3* (*IFIT3*), *IFIT2*, and *Oligoadenylate synthase like* (*OASL*) in the top 20 markers (fig. S5), and a small proportion of cells expressing interferon lambda. We noted that a small proportion of the cells were expressing antiviral genes in general after infection, consistent with previous studies (*5*, *11*). Notably, this proportion did not appreciably increase after infection (Fig. 4C), nor did the level of expression of antiviral genes per cell (fig. S6A), likely due to effective host antagonistic functions deployed by EVs (*22*).

Five host cell clusters contained infected cells. The antiviral clusters, 2 and 5, showed small numbers of infected cells. These mostly exhibited low viral RNA content, potentially representing early infection or infections limited or aborted by innate immune responses.

Clusters 4, 7, and 9 were enriched in highly infected cells, suggesting that these cell states may represent phenotypes occurring late in infection. Clusters 4 and 7 exhibited infection-specific behavior, characterized by two distinct stress responses. The cell phenotype represented by cluster 4, which is nearly absent from the mock-infected condition (Fig. 4A), exhibits markers associated with the integrated stress response (ISR) downstream of activating transcription factor 4 (*ATF4*), including increased expression of *ATF3* and DNA Damage Inducible Transcript 3 (*DDIT3*). This likely reflects a late infection phenotype when viral protein expression is peaking. *ATF4/ATF3*-mediated responses have been implicated in several positive-sense RNA viruses including the related EV, CV-B3. Besides regulating the ISR, ATF3 regulates cellular antiviral, apoptotic, and autophagy pathways via the inhibition of ISGs such as ISG15 (*23*–*25*). The presence of *Protein Phosphatase 1 Regulatory Subunit* 15*A* (*PPP1R15A*) [formerly *Growth Arrest And DNA Damage-Inducible Protein* (*GADD34*)] and *BCL2 Antagonist/Killer* 1 (*BAK1*) expression in cluster 4 (fig. S6B), both of which are expressed downstream ISR and the unfolded protein response, suggests that *ATF3* is mediating a proapoptotic response in these cells (*26*).

Cells in cluster 7 were characterized by the expression of a diverse set of genes, with many related to metabolic stress and mitochondrial dysfunction. The transcriptional phenotype of these cells appeared to shift in response to infection. To resolve expression differences at a finer resolution, we performed a targeted reanalysis of this subset of cells, yielding three subclusters: 7.0, 7.1, and 7.2. Subclusters 7.0 and 7.2 are present in both infected and uninfected conditions and are associated with the expression of genes involved in plasma membrane regulation and mitochondria-oxidative metabolism, respectively. Upon infection, a distinct phenotype (subcluster 7.1) emerged among infected cells that shared markers with the highly infected cluster 4, including *CITED2*, *FOS*, *TXNIP*, and *CCNG2* genes. However, in contrast to cluster 4, subcluster 7.1 appears to represent an antiapoptotic cell phenotype, due to the presence of *MTRNR2L12*, *TRIAP1*, and *RRM2B* expression (fig. S6C).

### Interpreting adaptive single-cell phenotypes

The combined observation of viral genotype and cell phenotype allows us to correlate the adaptation of viral populations with phenotypic changes in infected cells (Fig. 4A iii). We mapped our genotypes acquired from each passage onto the phenotypic maps of the captured cells obtained through conventional single-cell sequencing. Over passage, we observed a trend that genotypes predominating in later passages coalesce into two infection-specific clusters, 4 and 7, with the majority falling in cluster 4.

Within these five passages, two dominant genotypes emerged, both with multiple mutations in the capsid protein relative to the modal genotype in passage 1. On the basis of the viral read counts in each cell (Fig. 4D), we observe that the early genotypes, intermediate in the “subway map,” are associated with lower median viral RNA content at 6 hours postinfection (hpi) compared to genotypes arising later, suggesting that these later genotypes are reaching peak replication faster. Because the majority of the mutations in these later genotypes, the terminal nodes on the subway map (carrying non-synonymous substitutions: T210M, N847D + A1050V, H352Y + S848F, and I814V + S848F), lie on the capsid surface, we predict that these genotypes will exhibit enhanced entry or uncoating. The grouping of these genotypes together in the phenotypic cluster (*4*) characterized by markers of ER and proteotoxic stress associated with late infection further supports this interpretation (Fig. 4).

## DISCUSSION

Genotypic diversity is so much a part of viral populations that we often invoke specific terminology, such as quasi-species, to describe its emergent behaviors. In this work, we establish SEARCHLIGHT as a facile method to recover viral genotypes from infected samples by leveraging existing single-cell platforms to partition individual infected cells before cDNA synthesis, sequencing, and genotype reconstruction. This allows us to capture and assemble hundreds of complete, and near-complete, viral haplotypes from individual infected cells and place them within a genotypic network. Our analysis of the dynamics of the population as it navigates this network highlights the ability of viral populations to remain centered on a slowly moving modal genotype while exploring multiple mutational paths, with branches operating in parallel to reach adaptive genotypes many mutational steps away. Interpreting such genotype-phenotype relationships from single-population sequencing experiments is only possible with single-genome resolution.

One aspect of viral population dynamics illustrated by these genotype networks is how they span and spread across mutational landscapes, connecting distinct adaptive genotypes and spanning apparent “fitness valleys” in the local selective landscape. This is similar to the phenomenon known as “stochastic tunneling,” where populations reach adaptive genotypes through chains of low-frequency, less fit genotypes linked through mutations that drift into evolving populations (*27*, *28*). In our large population experiment, similar mutational “tunnels” in the central ring of our subway map connect nearby high fitness genotypes.

That the genotypes constituting these tunnels persist in the population over passage also highlights the ability of viral populations to hedge their evolutionary bets, similar to other theoretical and biological systems where mixed phenotypic strategies can optimize fitness in dynamic environments (*29*–*31*). Viral populations can carry tremendous diversity that represents a memory of past population diversity along with new mutations that, upon selection or bottleneck, can emerge or reemerge to dominance. These genotypes form a connected network that might provide robustness to changing immune and environmental pressures. Further in-depth study of the dynamics and interactions of genotypes within structured viral populations and how these genotype networks relate to the biophysical constraints of viral biology will clarify their significance in viral population dynamics.

It is notable that the shared sites of variability in EV-A71 and EV-D68, which were observed here in conditions of relaxed immune selection, overlap with those identified under antibody selection (*17*). It might suggest that these sites, which are polymorphic in both species, have evolved to be mutationally robust in response to past immune selection at this immunodominant site, in a sense, positioning drift and immune escape as parallel mutational forces as described for murine norovirus, a relative in the order Picornavirales (*32*) Moreover, most of the non-synonymous mutations that we observed in our genotypes were identified in previous studies of EV-A71 and EV-D68 adaptation in cultured cells or in vivo models (*33*, *34*).

One key advantage of our technique is the ability to directly link viral genotype to transcriptional phenotype without engineering cells or viruses. As such, we anticipate that our method will be compatible with off-the-shelf cell lines, primary cells, patient- and animal-derived samples, as well as engineered clones. This allows investigation of the evolution and adaptation of a virus in nearly natural environments with contemporary strains and complex viral populations. It is also compatible with other sequence capture methods or B and T cell receptor sequencing methods that use the same 5′ capture approach.

Several key limitations remain to be optimized in the SEARCHLIGHT procedure. Now, with the sequencing depth and RT primer concentrations, we have a limited ability to reconstruct full genomes, limiting our analysis here to the coding region, to avoid difficulties in sequencing the highly structured internal ribosomal entry site at the viral 5′ end. Our method does not sample deep enough in the viral population of each cell to make effective use of the UMIs for error correction of viral transcripts, so our analysis is limited to the cell-level consensus. The consensus genomes recovered in several cells included ambiguous nucleotides, indicating the presence of multiple genotypes, either from coinfecting particles or from newly emerging mutations. However, future advancements in single-cell technologies, deeper sequencing, and further bioinformatic development may enable the identification of viral subpopulations within individual cells. Moreover, the current resolution of SEARCHLIGHT may make it useful in exploring the dynamics of recombination in virus-infected cells. However, this is outside the scope of the current analysis.

This analysis reconstructs the structure of experimental populations to better understand how viral populations diversify and explore their mutational neighborhood to discover adaptive genotypes. Applying this method to the dynamics of immune and therapeutic pressure in vivo and in vitro will be of specific interest in understanding the repertoire of mutational pathways available to escape such pressures.

## MATERIALS AND METHODS

### Cell lines and viruses

#### ATCC cell line

RD [American Type Culture Collection (ATCC), CCL-136] and HeLa (ATCC, CCL-2) cells were maintained in Dulbecco’s modified Eagle medium (DMEM) (ATCC, 30-2002) supplemented with 10% fetal bovine serum (FBS) (Gibco) and 1× penicillin/streptomycin/glutamine (100× PSG stock, Gibco). Cells were grown at 37°C and 5% CO_2_.

#### Viruses

##### Long-term passage: EV-A71 (Tainan) and EV-D68 (MO/14-18947)

EV-A71 and EV-D68 were produced through a single amplifying passage (passage 1, “P1”) of the virus stock received from BEI Resources [catalog numbers NR-471 (Tainan/4643/1998) and NR-49129 (US/MO/14-18947)] in RD cells with 2.5% FBS DMEM, at 37° and 33°C, respectively. After 3 days and the observation of complete cytopathic effect (CPE), the cells were submitted to two rounds of freeze/thaw and centrifuged at 2000*g* for 5 min. The supernatants were collected and aliquoted into cryovials and stored at −80°C. For the passage experiment of EV-A71, the viruses were harvested at 24 hpi instead of 3 days. The passage history of EV-A71 and EV-D68 from BEI Resources [catalog numbers NR-49471 (Tainan/4643/1998) and NR-49129 (US/MO/14-18947)] was retrieved through personal communication with BEI personnel responsible for propagating these viruses: EV-A71 (NR-471) deposited material is from a second passage in RD cells and was passaged twice in RD cells at BEI during the growth of the most recent lot. The EV-A71 P1 modal sequence of the population differs from the cited database entry by three SNVs and a single non-synonymous substitution in the capsid, E to Q at position 710. EV-D68 (NR-49129) deposited material is fourth passage in RD cells, and it was passaged three times in RD cells at BEI during the growth of the most recent lot.

##### cDNA molecular clone: CV-B3 (Nancy strain)

The CV-B3 cDNA molecular clone (p53CB3T7) was provided by G. Belov, University of Maryland, College Park. For CV-B3 production, we produced viral RNA from the molecular clone using T7 RNA polymerase (New England Biolabs). These viral RNA were purified and transfected to HeLa cells using the TransIT-mRNA Transfection Kit (Mirus) at 37°C for 3 days to achieve complete CPE. We harvested the virus by freeze/thaw as described above to produce our P0 stock. The viruses from P0 were titrated by TCID_50_ assay and passed at a low MOI (0.1) in HeLa cells to recover P1, and this P1 stock was used for the subsequent single-cell experiment.

### Viral infection and cell preparation

RD cells were plated on the day before the experiment (~2 × 10^6^ cells in T-25 flasks). One extra flask was used to count the cells before to ensure the cells count for MOI calculation. Inoculums of 0.3 MOI were prepared fresh using in FBS-free medium of the three viruses (EV-A71, EV-D68, and CV-B3 P1). After removing the medium and washing with warm phosphate-buffered saline (PBS), the cells were incubated for 1 hour with the inoculums (rocked each 10 min) at 37°C for EV-A71 and CV- B3 and at 33°C for EV-D68. After 1 hour, the inoculum was removed, and 5% FBS medium was added. The cells were incubated into their respective temperatures (37° and 33°C) until the time point of collection (6 hpi). Mock-infected cells were also collected at 6 hpi. After 6 hpi, the cells were submitted to the standard protocol of sample preparation proposed by 10x Genomics [Chromium Single Cell 5′ Reagent Kits User Guide (v2 Chemistry Dual Index), document number CG00331 - Rev E, August 2022]. Briefly, the medium is removed, and the cells are washed with warm PBS. TrypLE is added (2 ml) to dissociate the cells, and 2 ml of medium is added to homogenize the cells. The cells are counted and checked for viability. After counting, cells are centrifuged at 500*g* for 5 min. Then, the supernatant is removed, and the cells are washed with 1× PBS with 0.04% bovine serum albumin (BSA) and centrifuged again at 500*g* for 5 min. Supernatant is removed, and the appropriate volume of PBS/BSA is added to achieve the concentration of cells desired (~1000 cells/μl). Cells are counted once more to assess cell viability, and cells are moved to ice before loading on the microfluidic controller.

### scRNA-seq cDNA library generation

All samples were calculated to achieve ~5000 to 8000 targeting cells in the single-cell preparations using a Chromium Next GEM Single-Cell 5′ standard kit. For the preparation of the cDNA and sequencing library generation, we followed the instructions from the user guide for the Chromium Next GEM Single Cell 5′ Reagent Kit v2 (Dual Index) with one modification. The modification consists of a primer spike-in approach during the RT master mix preparation step (step 1.1), similar to other applications, including CRISPR guide RNA-seq by Replogle and colleagues (*35*). In their method, they report the concentration of poly-dT RT primer as ~100 pmol in each RT reaction. In our approach, virus-specific primers tiled across the viral genome were added to the master mix along with poly-dT RT primer. The virus-specific primer concentration into each RT reaction was ~15 pmol each, and three to four primers were added depending on the virus primer set (table S1) (*36*). All the other reagents were added according to the protocol, except the nuclease-free water, which was adjusted to accommodate the spike-in volume. All other steps were followed to produce cDNA and subsequent Illumina sequencing libraries for conventional single-cell sequencing. The Illumina library preparation was submitted to quality control in the TapeStation D1000 high sensitivity for size distribution and DNA concentration was measured by a Qubit High Sensitivity dsDNA kit. The molar concentration of the libraries was determined, and the samples were diluted for sequencing according to the manufacturers’ protocols. Sequencing was designed to yield 50,000 reads per target cell.

### PCR for virus-specific single-cell sequencing

While the cDNA produced from the first step of the 10x single-cell pipeline is used for sequencing library preparation, only 50 ng is used for Illumina library prep. The remaining cDNA served as template for a virus-specific amplification by polymerase chain reaction (PCR) to enrich and increase the signal for virus-specific fragments. We performed the virus-specific reaction with a Kapa HiFi HotStart PCR kit, following the manufacturer’s instructions, and 25 cycles as recommended for high-fidelity amplification. Each primer set was performed in a separate reaction, with 10x cDNA Forward Primer (200089) as a common forward primer to retain single-cell barcoding information, with the virus-specific reverse primer of each fragment. After each PCR reaction, the amplicons were run into the TapeStation D5000 kit to visualize the size distribution, and the concentration was measured by a Qubit Broad Range dsDNA kit. Then, all products are pooled at equimolar concentrations and cleaned up with SPRI beads (left-side size selection, 0.6× ratio) to remove adaptors that can also be amplified during the PCR (<200 base pairs). The cleaned products are again measured by TapeStation and Qubit to verify purity and concentration of the amplicons. The amplicons were submitted to PacBio sequencing in the Frederick National Laboratory for Cancer Research (CCR-Sequencing Facility). Briefly, the amplicons were used as input for library preparation using a SMRTbell prep kit 3.0 and sequenced on PacBio Sequel II with one flow cell for each sample (targeting up to 4 million reads per sample).

### Viral genotype reconstruction and network

#### Anchovy

Anchovy is an approach wherein viral reads are mapped and binned into cell-specific bins, which are used to compute the consensus genotype in the cell. First, viral sequences are identified by mapping our PacBio amplicon sequencing reads, in the form of a FASTQ file, to the viral genome using minimap2 (*37*). As input, we have the FASTQ files from PacBio sequencing and the viral reference as a fasta. The output of minimap is the SAM file. We then run an in-house script, anchovy.py, which identifies the cell barcodes associated with bona fide cells identified by CellRanger analysis of the Illumina sequencing of our 10x libraries. The anchovy.py is a script that we developed to recover viral reads along with 10x barcodes from Nanopore and PacBio long-read sequencing to generate viral consensus sequences per cell (fig. S1). We used this script to generate a CSV file summarizing the barcodes and associated consensus, the aligned and consensus fasta files for each barcode. The consensus in each cell is determined using sam2consensus (*38*). Because of limitations in coverage in the highly structured UTR, we extracted the coding sequence of each viral sequence to focus on amino acid changes.

#### Cytoscape

Using the “_Annot.csv” files for each sample, a list of binary interactions was generated in R by determining genotypes separated by SNVs. This table of source and target nodes, along with annotations of mutation distance and genotype information, was used as input for visualization in Cytoscape 3.9.1 (*39*).

### scRNA analysis

#### CellRanger/Seurat

10x Genomics CellRanger 7.0.0 was performed on all single-cell libraries produced, using the Binary Base Call (BCL) files from Illumina sequencing output: MakeFastQ, CellRanger Count, and CellRanger Aggregate functions (*40*). The final outputs of count were barcodes.tsv, features.tsv, and matrix.mtx (all from filtered output). All these outputs were used as input in Seurat (R toolkit for single-cell analysis) for further analysis. Using Seurat V4, we proceeded with preprocessing of the data (quality control and normalization), linear dimensionality reduction, clustering, uniform manifold approximation projection (UMAP), differentially expressed genes, and the aggregation of the samples from the pertinent experiments together (*41*). All embeddings of UMAPs generated were exported for the generation of Fig. 4 and fig. S4. Biological interpretation of each cluster used DAVID (*42*, *43*) and manual curation of the gene markers returned by the Seurat FindAllMarkers function. These UMAP embeddings were also used to integrate the data from the transcriptome with the viral haplotypes for a full picture of the SEARCHLIGHT output, the viral genotype in the phenotypic space.
