# Supplementary material for: Structure and dynamics of enterovirus genotype networks
Source: Sci Adv. 2024 Jun 19;10(25):eado1693. doi: 10.1126/sciadv.ado1693 (PMC11186490; doi:10.1126/sciadv.ado1693)
Supplement: Supplementary file 1 — Figs. S1 to S6 Tables S1 and S2 [file sciadv.ado1693_sm.pdf]

Science Advances
AAAS

Supplementary Materials for

Structure and dynamics of enterovirus genotype networks

Nathânia Dábilla and Patrick T. Dolan

Corresponding author: Patrick T. Dolan, patrick.dolan@nih.gov

Sci. Adv. **10**, eado1693 (2024)
DOI: 10.1126/sciadv.ado1693

This PDF file includes:

Figs. S1 to S6
Tables S1 and S2

Figure S1

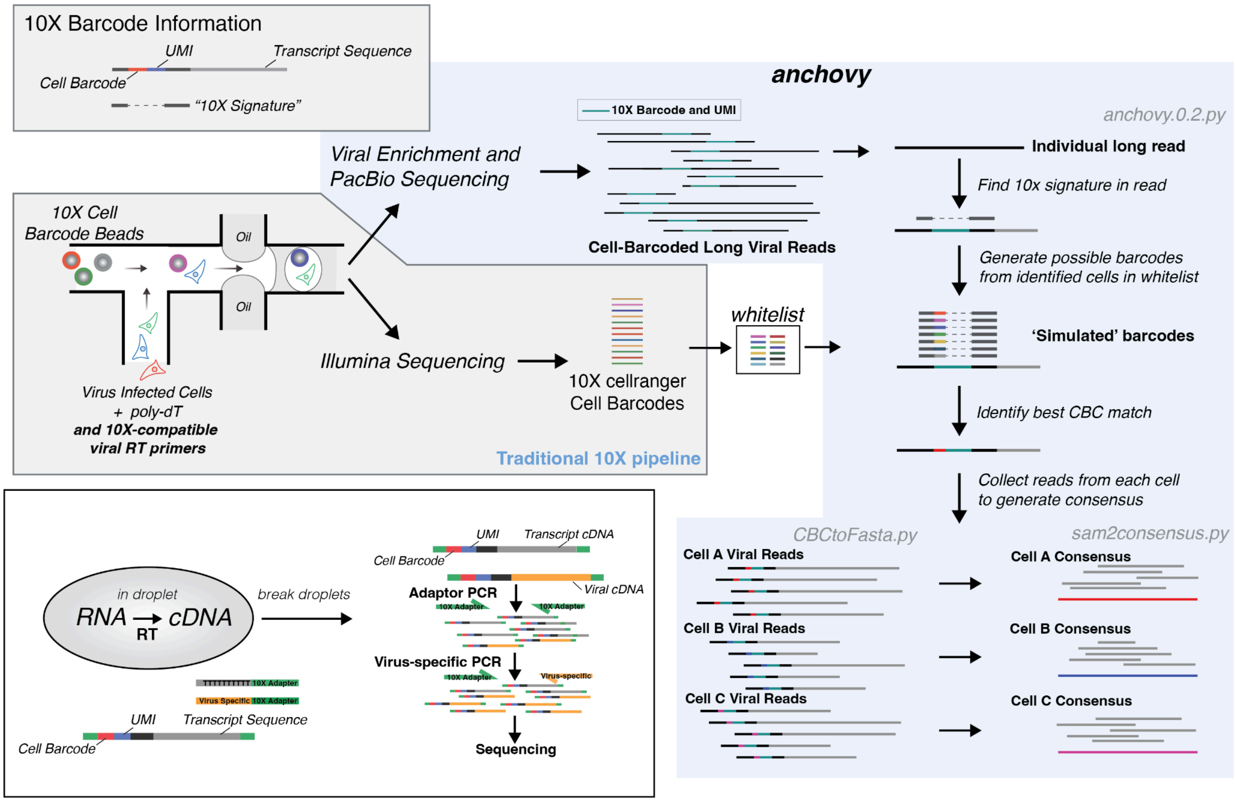

10X Barcode Information
UMI
Transcript Sequence
Cell Barcode
"10X Signature"
anchovy
10X Barcode and UMI
anchovy.0.2.py
Viral Enrichment and PacBio Sequencing
Individual long read
Find 10x signature in read
Cell-Barcoded Long Viral Reads
Generate possible barcodes from identified cells in whitelist
10X Cell Barcode Beads
Oil
Oil
whitelist
'Simulated' barcodes
Illumina Sequencing
10X cellranger Cell Barcodes
Virus Infected Cells + poly-dT and 10X-compatible viral RT primers
Identify best CBC match
Traditional 10X pipeline
Collect reads from each cell to generate consensus
CBCtoFasta.py
sam2consensus.py
Cell A Viral Reads
Cell A Consensus
Cell B Viral Reads
Cell B Consensus
Cell C Viral Reads
Cell C Consensus
UMI
Transcript cDNA
Cell Barcode
Viral cDNA
Adaptor PCR
in droplet
RNA → cDNA
RT
break droplets
Virus-specific PCR
UMI
Transcript Sequence
Cell Barcode
Sequencing

Fig. S1.

Anchovy pipeline. cDNA generated by 10x Genomics is sequenced by conventional illumina sequencing and analyzed by the CellRanger pipeline to yield the associated matrix, barcode, and gene files. The cDNA is also amplified by fragment-specific primers to yield 10x-barcoded amplicon pools that covers the full genome. The 10x barcode is identified in each read, and the cell barcode is matched to one of the barcodes identified in the CellRanger pipeline. This assures that the droplets carrying viral genome information are also 'real' cells. The viral reads associated with each cell barcode are used to generate a consensus sequence for each cell.

Figure S2

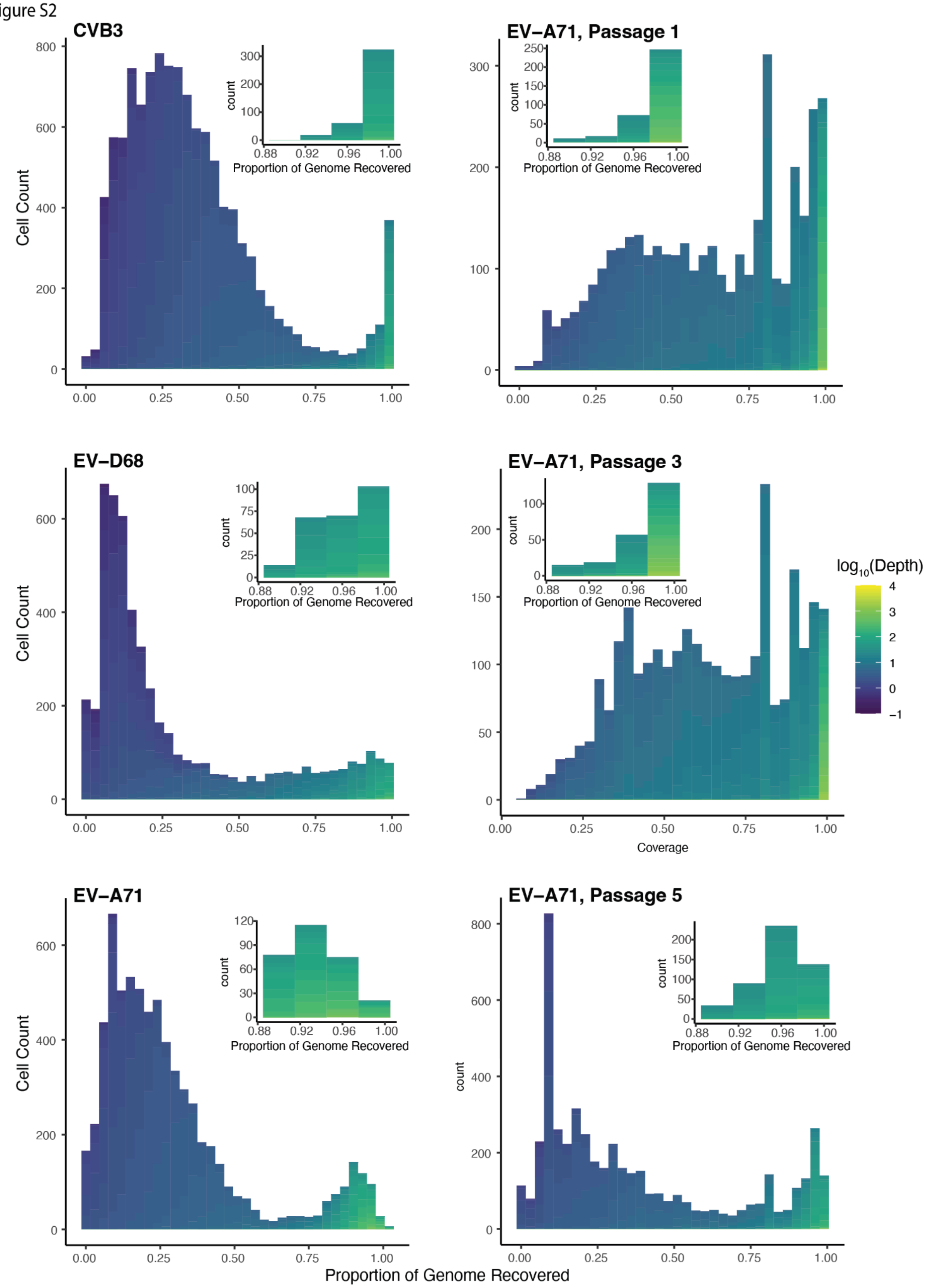

CVB3
EV−A71, Passage 1
EV−D68
EV−A71, Passage 3
EV−A71
EV−A71, Passage 5
Cell Count
count
Proportion of Genome Recovered
Coverage
log10(Depth)

Fig. S2.

Histogram of the proportion of viral genome captured in each cell during SEARCHLIGHT sequencing experiments. Fill color represents the average depth of coverage across the viral genome in each cell. The inset shows the cells from which consensus haplotypes were recovered for subsequent analysis.

Figure S3

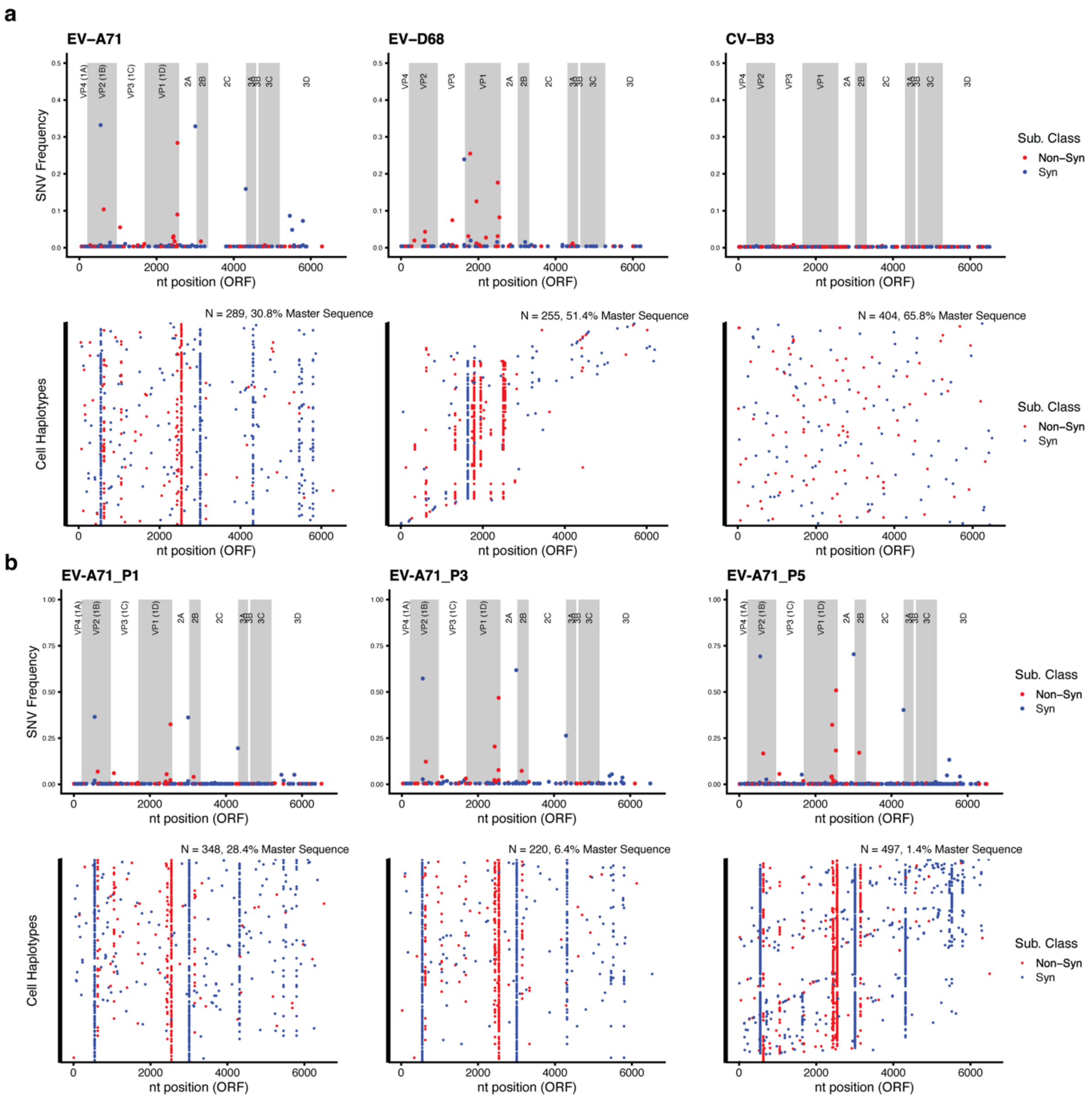
a
EV−A71
EV−D68
CV−B3
SNV Frequency
nt position (ORF)
Sub. Class
Non−Syn
Syn
N = 289, 30.8% Master Sequence
N = 255, 51.4% Master Sequence
N = 404, 65.8% Master Sequence
Cell Haplotypes
b
EV-A71_P1
EV-A71_P3
EV-A71_P5
N = 348, 28.4% Master Sequence
N = 220, 6.4% Master Sequence
N = 497, 1.4% Master Sequence
VP4 (1A)
VP2 (1B)
VP3 (1C)
VP1 (1D)
2A
2B
2C
3A
3B
3C
3D

Fig. S3.

The ‘bulk’ view of population diversity (top) and the ‘phased’ haplotype alignment (bottom). In the haplotype alignment, each haplotype derived from an individual cell is shown as a single row, with differences from the modal genotype shown as points. Color indicates non-synonymous or synonymous mutations. Viral proteins are labeled and denoted by alternate shading. **(A)** First row refers to the strains Enterovirus-A71 (strain Tainan), Enterovirus-D68 (strain MO/14-18947), and Coxsackievirus B3 (strain Nancy) belonging to three distinct enterovirus species (A, B, and D). **(B)** Second row refers to Enterovirus-A71 passage1, passage 3 and passage 5.

Figure S4

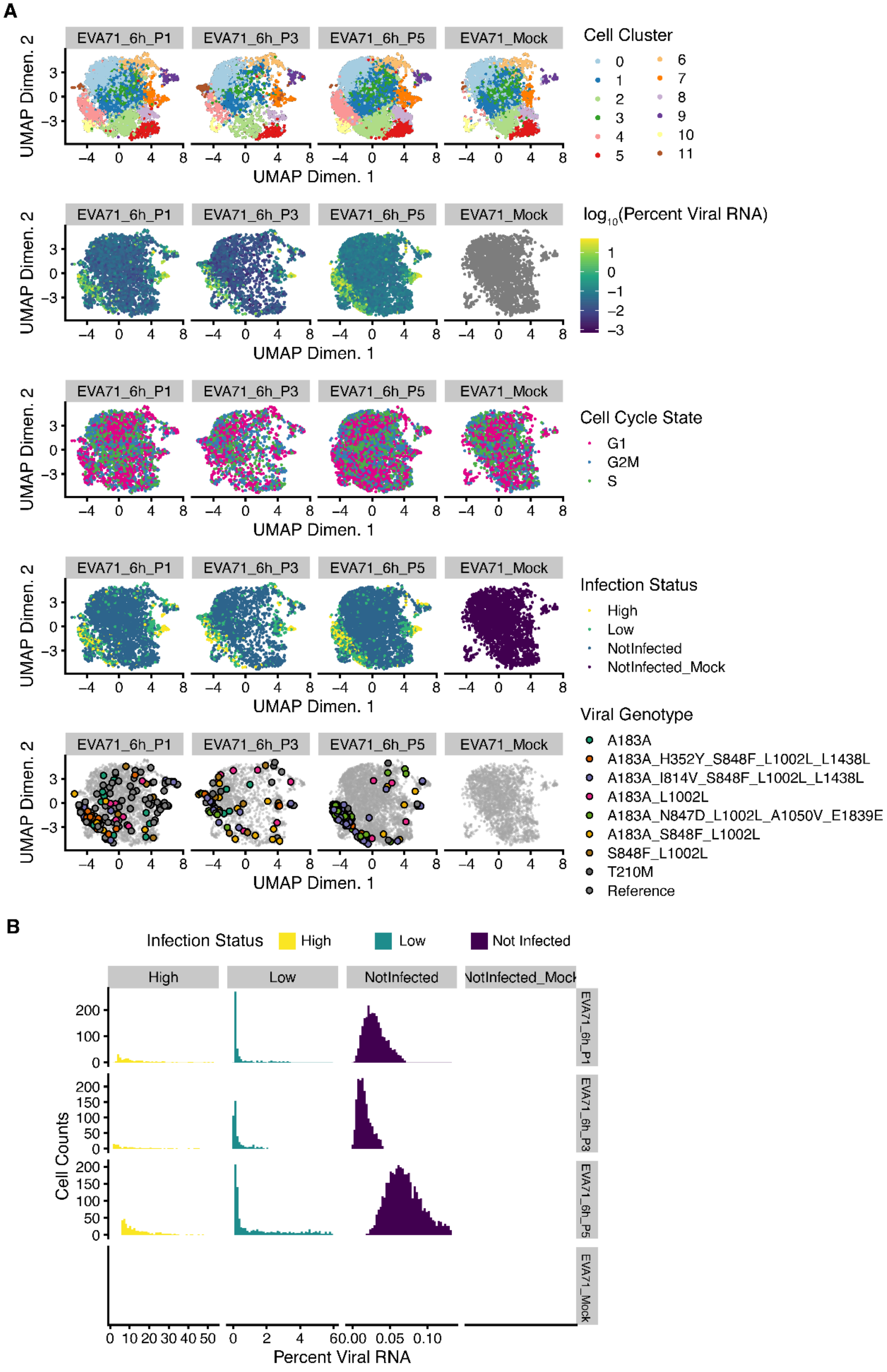
A
EVA71_6h_P1
EVA71_6h_P3
EVA71_6h_P5
EVA71_Mock
UMAP Dimen. 1
UMAP Dimen. 2
Cell Cluster
0 1 2 3 4 5 6 7 8 9 10 11
log10(Percent Viral RNA)
Cell Cycle State
G1
G2M
S
Infection Status
High
Low
NotInfected
NotInfected_Mock
Viral Genotype
A183A
A183A_H352Y_S848F_L1002L_L1438L
A183A_I814V_S848F_L1002L_L1438L
A183A_L1002L
A183A_N847D_L1002L_A1050V_E1839E
A183A_S848F_L1002L
S848F_L1002L
T210M
Reference
B
Infection Status
High
Low
Not Infected
High
Low
NotInfected
NotInfected_Mock
EVA71_6h_P1
EVA71_6h_P3
EVA71_6h_P5
EVA71_Mock
Cell Counts
Percent Viral RNA

Fig. S4.

Connecting genotype with infected cell phenotype. **(A)** Uniform Manifold-Assisted Projection (UMAP) of the cell transcriptional phenotypes recovered from the passaged cell populations. (i) Clustering of transcriptional phenotypes derived from captured cells. (ii) Percent of viral RNA in each cell. (iii) Cell Cycle State of each cell. (iv) Identification of infected and uninfected cells. Cells are also classified by high- and low-viral RNA content. (v) Major genotypes captured by SEARCHLIGHT placed onto the phenotypic map of the host cells. **(B)** Histogram of the percent of viral RNA captured in each cell according to each Infection Status group, yellow for High, green for Low and dark purple for Not infected.

Figure S5

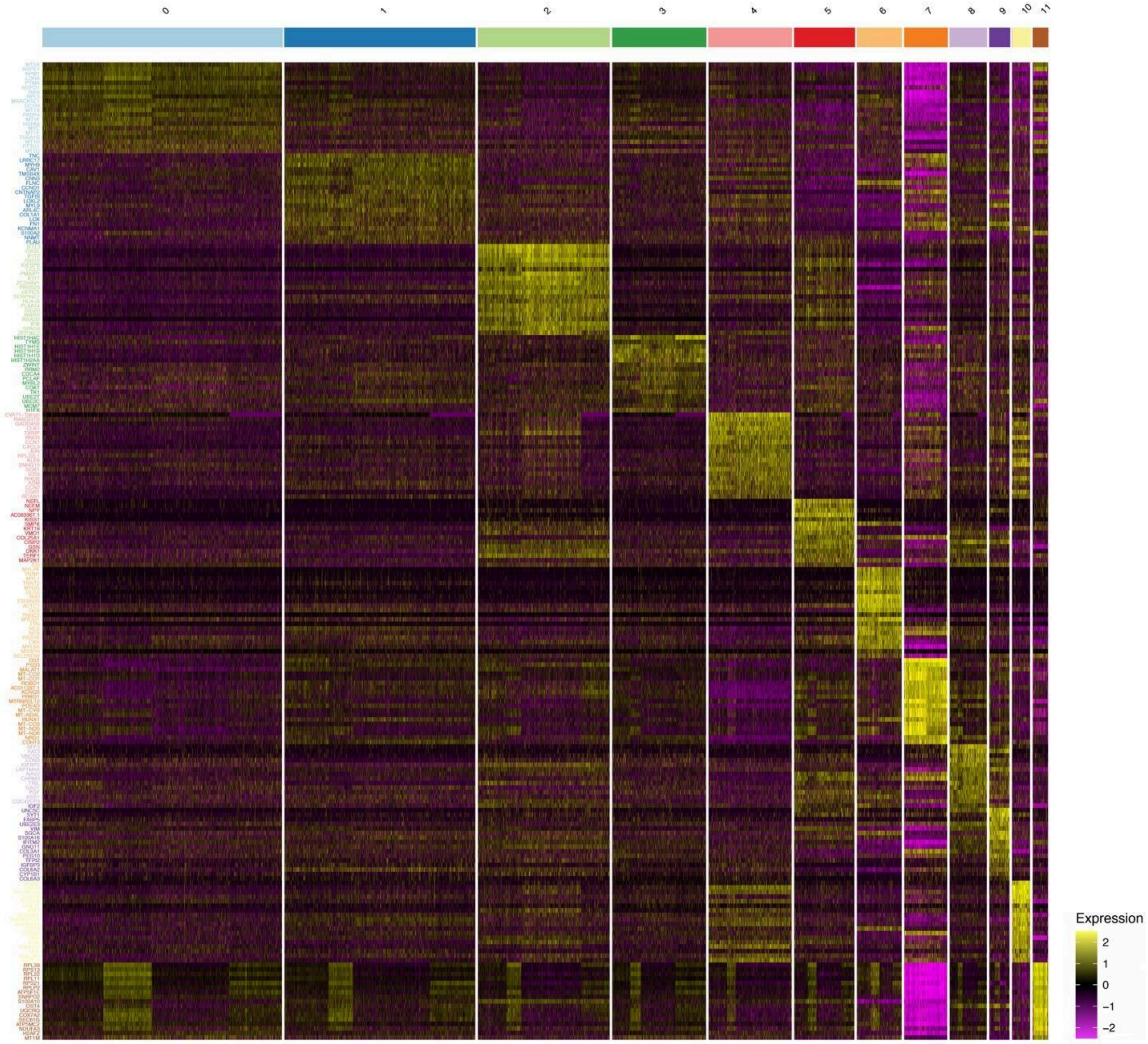

Fig. S5.

Heatmap of top20 marker genes of each cluster. The genes label are colored according to the clusters colors.

Figure S6

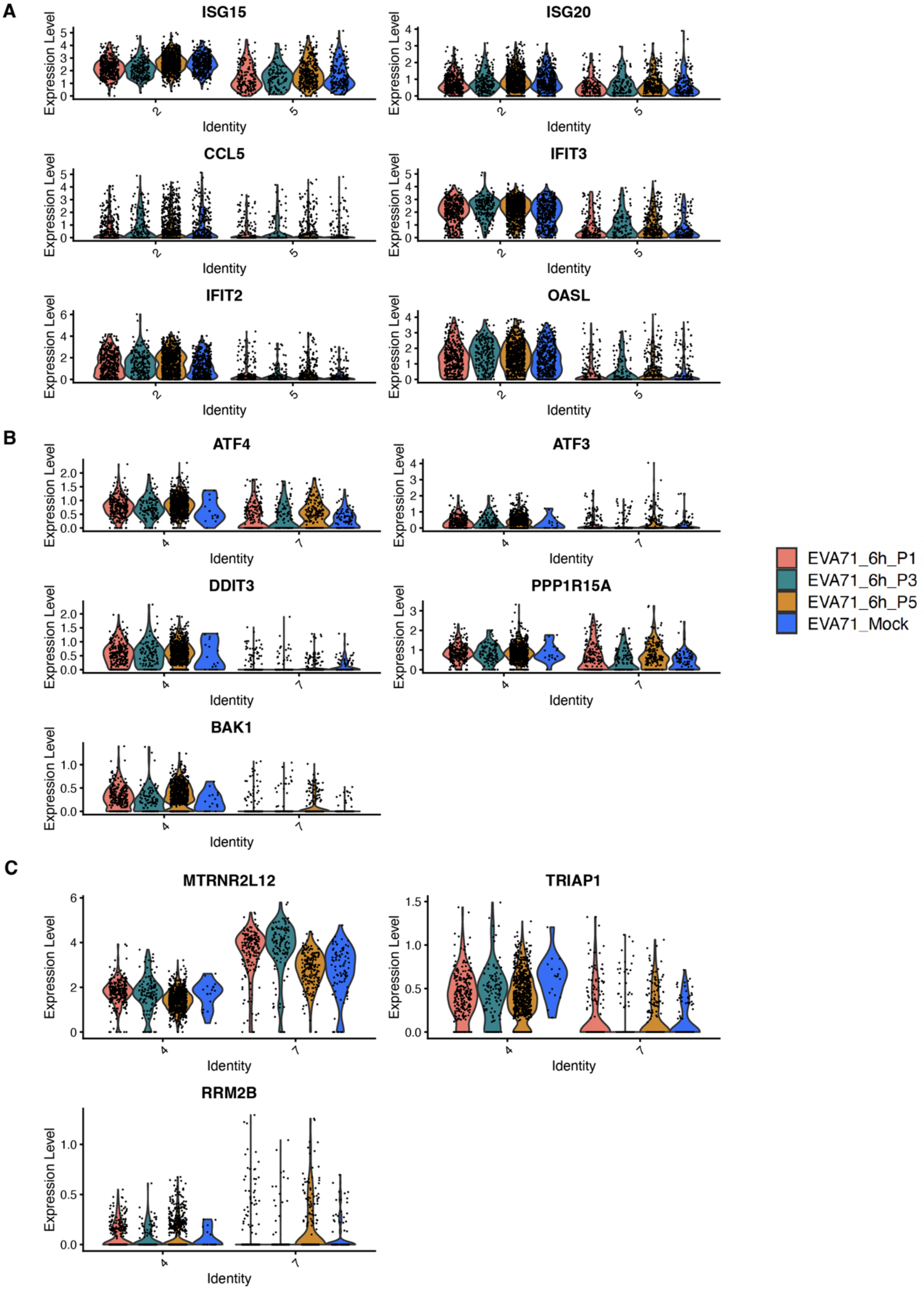
A
ISG15
ISG20
CCL5
IFIT3
IFIT2
OASL
B
ATF4
ATF3
DDIT3
PPP1R15A
BAK1
C
MTRNR2L12
TRIAP1
RRM2B
Expression Level
Identity
EVA71_6h_P1
EVA71_6h_P3
EVA71_6h_P5
EVA71_Mock

Fig. S6.

Violin plot of Expression Level of markers genes per cluster per condition. **(A)** Expression Levels of interferon-stimulated genes (ISGs) in cluster 2 and cluster 5. **(B)** Expression Levels of Integrated Stress Response (ISR) and pro-apoptosis markers. **(C)** Expression Levels of anti-apoptosis markers.

Primers with 10x genomics adaptors

name	seq
CVB3_4_R_10X	5'-AAGCAGTGGTATCAACGCAGAGTACGCCCTGCTAAACGTAACCCATT-3'
CVB3_9_R_10X	5'-AAGCAGTGGTATCAACGCAGAGTACCTTCACTCCCTGTTCCATTGCA-3'
CVB3_14_R_10X	5'-AAGCAGTGGTATCAACGCAGAGTACAAGCATTCTCTTGGTGGGTGTG-3'
EVD68_MO_15_R_10X	5'-AAGCAGTGGTATCAACGCAGAGTACTGTGACTTCAATGTCCACGTCA-3'
EVD68_MO_12_R_10X	5'-AAGCAGTGGTATCAACGCAGAGTACAGTGAACTCTCTCCAATTAGCATATCT-3'
EVD68_MO_6_R_10X	5'-AAGCAGTGGTATCAACGCAGAGTACACCAATGAGTGTAATATCTAGATATGTTTCC-3'
EVA71_NS_4_R_10X	5'-AAGCAGTGGTATCAACGCAGAGTACTGACGATCTGTTGTTTGTATCCGT-3'
EVA71_NS_9_R_10X	5'-AAGCAGTGGTATCAACGCAGAGTACAACTCGTCGGGTTCATGAAGC-3'
EVA71_Cap_9R_10X	5'-AAGCAGTGGTATCAACGCAGAGTACTCCAACTAATCCTGCTCTGCTG-3'
EVA71_Cap_1R_10X	5'-AAGCAGTGGTATCAACGCAGAGTACGCCAATAGTTAATTGTGCCACCC-3'
EVA71_Cap_3R_10X	5'-AAGCAGTGGTATCAACGCAGAGTACGGTGTTGCAATTCGAAGCCATC-3'

Primers without 10x genomics for virus-specific PCR enrichment

cDNA Primers - part number 2000089
From Chromium Next GEM Single Cell 5' Reagent Kit v2 (Dual index), CG000331, Rev E, August 2022

cDNA Forward Primer:
5'-CTACACGACGCTCTTCCGATCT-3'

CVB3_4_R	5'-GCCCTGCTAAACGTAACCCATT-3'
CVB3_9_R	5'-CTTCACTCCCTGTTCCATTGCA-3'
CVB3_14_R	5'-AAGCATTCTCTTGGTGGGTGTG-3'
CVB3_18_R	5'-CGAATGCGGAGAATTTACCCCT-3'
EVD68_MO_15_R	5'-TGTGACTTCAATGTCCACGTCA-3'
EVD68_MO_12_R	5'-AGTGAACTCTCTCCAATTAGCATATCT-3'
EVD68_MO_6_R	5'-ACCAATGAGTGTAATATCTAGATATGTTTCC-3'
EVA71_NS_4_R	5'-TGACGATCTGTTGTTTGTATCCGT-3'
EVA71_NS_9_R	5'-AACTCGTCGGGTTCATGAAGC-3'
EVA71_Cap_9R	5'-TCCAACTAATCCTGCTCTGCTG-3'
EVA71_Cap_1R	5'-GCCAATAGTTAATTGTGCCACCC-3'
EVA71_Cap_3R	5'-GGTGTTGCAATTCGAAGCCATC-3'

Table S1.
Primers for SEARCHLIGHT and for virus-specific PCR enrichment of CV-B3, EV-D68_MO and EV-A71.

Cluster ID	Category	Term	Count	Enrichment Score	P-value	Genes
0	-	-	-	-	-	-
1	REACTOME_PATHWAY	R-HSA-1474244~Extracellular matrix organization	5	3.40720357	1.22E-03	COL15A1, COL5A1, LOX, THBS1, LOXL2
2	GOTERM_BP_DIRECT	GO:0051607~defense response to virus	18	10.36382823	3.16E-13	IFITM1, RSAD2, STAT2, IFIT1, SAMHD1, PARP9, IFIT3, IFIT2, OASL, IFIH1, BST2, ISG20, HERC5, ZNFX1, OAS1, OAS2, IRF1, DHX58
	REACTOME_PATHWAY	R-HSA-1169410~Antiviral mechanism by IFN-stimulated genes	6	2.904690624	8.13E-03	HERC5, OAS1, OAS2, IFIT1, USP18, OASL
3	REACTOME_PATHWAY	R-HSA-2555396~Mitotic Metaphase and Anaphase	18	10.35122472	2.75E-15	CDCA5, CDCA8, NDC80, ZWINT, AURKB, CENPF, CENPK, NUF2, CDK1, CENPM, BIRC5, KIF2C, CENPN, MAD2L1, SPC25
	REACTOME_PATHWAY	R-HSA-69242~S Phase	13	8.1734063	4.22E-11	GINS2, CDT1, FEN1, PCNA, UBE2C, RFC2, CDCA5, PRIM1, ESCO2, CCNA2, TFDP1, CDC45, MCM4
	INTACT	8354:H3 clustered histone 11(H3C11)	15	8.085331738	8.22E-09	HELLS, FEN1, RNASEH2A, RFC2, PRIM1, HMGB2, KIF11, MKI67, SHCBP1, TPX2, KIFC1, NUSAP1, MCM4, KIF2C, ASF1B
	REACTOME_PATHWAY	R-HSA-453276~Regulation of mitotic cell cycle	6	3.692159018	9.82E-05	CCNA2, UBE2C, CDK1, FBXO5, AURKB, MAD2L1
	REACTOME_PATHWAY	R-HSA-73894~DNA Repair	9	3.548156195	3.87E-04	CCNA2, RAD51AP1, FEN1, PCNA, CENPX, RFC2, TIMELESS, PCLAF, CLSPN
	BIOGREACTOME_PATHWAYRID_INTERACTION	R-HSA-4615885~SUMOylation of DNA replication proteins	5	2.514526497	9.41E-05	TOP2A, PCNA, CDCA8, BIRC5, AURKB
4	GOTERM_CC_DIRECT	GO:0090575~RNA polymerase II transcription factor complex	6	2.705178428	5.15E-05	NFYB, DDIT3, MAFF, FOS, ATF3, ATF4
	REACTOME_PATHWAY	R-HSA-74160~Gene expression (Transcription)	15	2.4509228	3.17E-03	CCNT2, BRF2, NFYB, CITED2, IGFBP3, SERPINE1, FOS, GEM, CCNG2, DDIT3, ZNF625, TXNIP, CCN2, SGK1, SRSF6
	BIOGRID_INTERACTION	4780:NFE2 like bZIP transcription factor 2(NFE2L2)	5	2.254218519	1.22E-03	DUSP1, DDIT3, MAFF, ATF3, ATF4
	REACTOME_PATHWAY	R-HSA-187037~Signaling by NTRK1 (TRKA)	5	2.230622153	1.31E-03	EGR1, RIT1, NRAS, FOS, SGK1
	GOTERM_BP_DIRECT	GO:0070059~intrinsic apoptotic signaling pathway in response to endoplasmic reticulum stress	4	2.12490232	2.68E-04	PPP1R15A, DDIT3, BAK1, ATF4
5	GOTERM_BP_DIRECT	GO:0009615~response to virus	9	5.673760667	1.84E-08	ISG20, IFIH1, IFITM1, OAS1, CCL5, IFIT1, IFIT3, IFIT2, OASL
6	GOTERM_BP_DIRECT	GO:0006937~regulation of muscle contraction	3	2.772216685	6.97E-03	TNNT2, TNNC1, TNNC2
	GOTERM_BP_DIRECT	GO:0005861~troponin complex	4	2.18897663	5.70E-05	TNNT2, TNNC1, TNNC2, TNNI1
7.0	REACTOME_PATHWAY	R-HSA-5620912~Anchoring of the basal body to the plasma membrane	7	2.380252937	3.04E-04	IQCB1, CEP162, CEP250, CEP83, NINL, SFI1, SDCCAG8
	REACTOME_PATHWAY	R-HSA-913709~O-linked glycosylation of mucins	5	2.278465092	2.99E-03	ST6GAL1, GALNT14, ST6GALNAC3, GALNT10, ST3GAL3
	INTACT	26037:signal induced proliferation associated 1 like 1(SIPA1L1)	7	2.259865363	1.46E-03	MAGI1, DENND1A, PPM1H, GAB2, MYO19, NINL, CEP128
	INTACT	23022:palladin, cytoskeletal associated protein(PALLD)	5	2.179038409	3.55E-04	CEP162, CEP83, MYO19, NINL, CEP128
7.1	REACTOME_PATHWAY	R-HSA-74160~Gene expression (Transcription)	81	14.53736992	6.94E-16	ZNF770, ZNF570, BTG1, DYRK2, MSTN, CITED2, ZNF490, TRIAP1, BMI1, ZFP30, ZNF568, ZNF564, ZNF442, YWHAH, ZNF441, ZNF561, PRKAB2, ZFP1, ZNF12, FOS, ZNF14, SIRT1, ZNF92, RRM2B, RRAGC, TXNIP, ZNF711, ZNF433, ZNF597, ZNF432, ZNF430, ZNF28, ZNF791, ZNF20, ZNF709, ZNF627, ZNF625, ZNF548, ZNF669, ZNF426, ZNF304, ZNF468, ZNF224, ZNF543, SKIL, SOCS4, ZNF267, ZNF264, CDKN2B, JUN, ZNF383, YES1, TAF13, NR1D2, HIPK1, KLF4, S[illegible]
	BIOGRID_INTERACTION	653220:X antigen family member 1A(XAGE1A)	6	2.651677107	1.18E-03	RYBP, ZC3H11A, ZBTB10, THAP12, ZBTB33, ZNF574
	INTACT	8339:H2B clustered histone 8(H2BC8)	5	2.055377609	8.80E-03	ELK4, RYBP, BCL10, BMI1, SIRT1
7.2	KEGG_PATHWAY	hsa05022:Pathways of neurodegeneration - multiple diseases	24	17.74401149	6.97E-15	COX8A, NDUFB9, NDUFA13, NDUFB8, NDUFA11, NDUFB11, NDUFA4, COX7A2, COX5A, ATP5F1E, TUBA4A, UQCRH, COX6B1, PSMD8, PSMB6, TUBA1C, TUBB3, UQCRQ, PSMA2, PSMB3, NDUFS5, ATP5PO, CYC1, SLC25A5
	KEGG_PATHWAY	hsa00190:Oxidative phosphorylation	16	12.30478641	4.58E-15	COX8A, NDUFB9, NDUFA13, NDUFB8, NDUFA11, NDUFB11, NDUFA4, COX7A2, COX5A, ATP5F1E, UQCRH, COX6B1, UQCRQ, NDUFS5, ATP5PO, CYC1
	BIOGRID_INTERACTION	4723:NADH:ubiquinone oxidoreductase core subunit V1(NDUFV1)	15	9.647794294	3.64E-12	NDUFB9, NDUFA13, NDUFB8, NDUFA11, NDUFB11, NDUFA4, MDH2, COX7A2, COX5A, UQCRH, COX6B1, UQCRQ, C1QBP, NDUFS5, CYC1
	GOTERM_BP_DIRECT	GO:0045333~cellular respiration	8	7.581074253	4.74E-10	COX8A, UQCRQ, NDUFA4, COX7A2, CYC1, COX5A, UQCRH, COX6B1
	MINT	55967:NADH:ubiquinone oxidoreductase subunit A12(NDUFA12)	8	5.340178393	2.38E-08	NDUFB9, NDUFA13, NDUFB8, NDUFA11, NDUFA4, NDUFB11, NDUFS5, UQCRH
	REACTOME_PATHWAY	R-HSA-1266738~Developmental Biology	18	5.074905535	2.10E-03	RPL21, RPL36A, RHOC, TUBA4A, RPL7, PSMD8, PSMB6, TUBA1C, RPS17, TUBB3, ARPC3, PSMA2, PSMB3, CDK4, RPL27A, AP2S1, PSME2, RPS11
	REACTOME_PATHWAY	R-HSA-9010553~Regulation of expression of SLITs and ROBOs	11	5.074754342	1.55E-07	PSMD8, PSMB6, RPS17, RPL21, PSMA2, PSMB3, RPL27A, RPL36A, PSME2, RPS11, RPL7
	GOTERM_CC_DIRECT	GO:0005751~mitochondrial respiratory chain complex IV	5	4.05584236	3.01E-06	COX8A, NDUFA4, COX7A2, COX5A, COX6B1
	REACTOME_PATHWAY	R-HSA-8852276~The role of GTSE1 in G2/M progression after G2 checkpoint	8	3.50385963	6.35E-07	PSMD8, PSMB6, TUBA1C, PSMA2, PSMB3, TUBB3, PSME2, TUBA4A
	BIOGRID_INTERACTION	196294:inner mitochondrial membrane peptidase subunit 1(IMMP1L)	7	3.335345797	6.01E-04	NDUFB9, NDUFA13, NDUFB8, MDH2, NDUFS5, CYC1, COX5A
	REACTOME_PATHWAY	R-HSA-192823~Viral mRNA Translation	6	3.306334435	2.97E-04	RPS17, RPL21, RPL27A, RPL36A, RPS11, RPL7
	BIOGRID_INTERACTION	6168:ribosomal protein L37a(RPL37A)	9	3.104649086	5.40E-05	BTF3, RPS17, RPL21, EIF6, EIF3I, RPL27A, SLC25A5, RPS11, RPL7
	REACTOME_PATHWAY	R-HSA-162909~Host Interactions of HIV factors	8	2.754824684	2.12E-05	PSMD8, PSMB6, RANBP1, PSMA2, PSMB3, AP2S1, PSME2, SLC25A5
	GOTERM_CC_DIRECT	GO:0000502~proteasome complex	6	2.730393717	3.81E-06	PSMD8, PSMB6, PSMA2, PSMB3, HSPB1, PSME2
	BIOGRID_INTERACTION	64976:mitochondrial ribosomal protein L40(MRPL40)	6	2.487082212	5.28E-04	GADD45GIP1, COX8A, MDH2, C1QBP, RPL27A, HSPB1
	MINT	4724:NADH:ubiquinone oxidoreductase subunit S4(NDUFS4)	4	2.300174265	5.40E-04	NDUFA13, NDUFB8, NDUFA11, NDUFS5
	GOTERM_CC_DIRECT	GO:0005750~mitochondrial respiratory chain complex III	3	2.218414983	1.44E-03	UQCRQ, CYC1, UQCRH
	BIOGRID_INTERACTION	2280:FKBP prolyl isomerase 1A(FKBP1A)	5	2.166625852	3.61E-04	HINT1, GPX4, PEBP1, TAGLN2, PPIB
	GOTERM_BP_DIRECT	GO:0071248~cellular response to metal ion	3	2.100712454	1.36E-03	MT1F, MT1G, MT1H
	MINT	22843:protein phosphatase, Mg2+/Mn2+ dependent 1E(PPM1E)	3	2.062654271	2.59E-03	SSR4, NDUFA4, SLC25A5
8	-	-	-	-	-	-
9	REACTOME_PATHWAY	R-HSA-2022090~Assembly of collagen fibrils and other multimeric structures	13	7.961709947	1.21E-10	COL18A1, COL15A1, COL11A1, COL12A1, LOXL3, PCOLCE, BMP1, COL5A1, LOX, COL5A3, PXDN, COL6A3, COL8A1
	GOTERM_MF_DIRECT	GO:0030020~extracellular matrix structural constituent conferring tensile strength	8	5.274300202	2.69E-06	COL18A1, COL15A1, COL5A1, COL11A1, COL5A3, COL12A1, COL6A3, COL8A1
	BIOGRID_INTERACTION	1667:defensin alpha 1(DEFA1)	10	4.546353292	5.07E-06	MOXD1, NOTCH3, COL18A1, ADAM17, BMP1, LAMB2, PXDN, IGFBP2, TIMP3, SLIT2
	INTACT	5636:phosphoribosyl pyrophosphate synthetase associated protein 2(PRPSAP2)	8	3.257857019	7.40E-06	PRPSAP1, ENDOV, SOCS1, ABI1, FYN, PRKCA, PRKD1, PTPN12
	REACTOME_PATHWAY	R-HSA-1638091~Heparan sulfate/heparin (HS-GAG) metabolism	6	2.500672606	1.86E-03	EXT1, VCAN, GPC1, SDC2, SDC3, BGN
	REACTOME_PATHWAY	R-HSA-8874081~MET activates PTK2 signaling	5	2.284272303	1.12E-03	ITGB1, COL5A1, LAMB2, COL11A1, COL5A3
	INTACT	2332:fragile X messenger ribonucleoprotein 1(FMR1)	12	2.233232239	5.84E-03	CLIP3, TRIM9, SYT1, PRKAR1A, SYT11, KIF5C, GFPT1, UBE2O, CXXC5, CDC42BPA, FBXO11, LRP1B
	REACTOME_PATHWAY	R-HSA-3656237~Defective EXT2 causes exostoses 2	4	2.102610638	1.18E-03	EXT1, GPC1, SDC2, SDC3
	GOTERM_BP_DIRECT	GO:0043567~regulation of insulin-like growth factor receptor signaling pathway	3	2.100221759	4.90E-03	IGFBP5, IGFBP3, IGFBP2
10	REACTOME_PATHWAY	R-HSA-187037~Signaling by NTRK1 (TRKA)	15	6.146698878	5.77E-07	EGR1, ATF2, SHC2, DUSP3, EGR4, RRAD, IRS2, FOS, FOSL1, RIT1, ARC, VGF, FOSB, TRIB1, JUNB
	GOTERM_MF_DIRECT	GO:0005484~SNAP receptor activity	7	2.66760694	1.94E-04	STX12, GOSR2, STX18, BNIP1, STX5, STX3, STX1A
	REACTOME_PATHWAY	R-HSA-74160~Gene expression (Transcription)	53	2.096724321	5.71E-03	ATF2, CDKN1A, CCNT2, ELL, CITED2, SERPINE1, BBC3, SNAPC1, SESN2, EPC1, SOX9, JUNB, YAF2, SAP30BP, HUS1, FOS, GTF2F2, PRKAB1, GEM, RRAGC, ZC3H11A, DDIT3, DDIT4, TXNIP, SRSF6, SLU7, ZFPM1, CEBPB, ZNF274, ZNF394, RRN3, RNMT, FOXO3, HDAC9, RELA, SKIL, ZNF267, PLK3, CDKN2B, TAF12, NFATC2, TNFRSF10B, GTF2H1, KLF4, ELL2, VEGFA, CDK7, POLR3C, TADA3, INTS6, ZNF655, LAMTOR3, ZNF697
	INTACT	5977:double PHD fingers 2(DPF2)	9	2.023013478	1.22E-03	KLF5, IRF1, FOS, ZNF655, SOX9, KLF4, ETS1, RELA, RELB
11	GOTERM_BP_DIRECT	GO:0045926~negative regulation of growth	5	7.632651158	6.43E-11	MT1A, MT1M, MT1G, MT1H, MT1B
	REACTOME_PATHWAY	R-HSA-5660526~Response to metal ions	5	7.632651158	2.05E-10	MT1A, MT1M, MT1G, MT1H, MT1B

Table S2.
Cluster markers enrichment analysis by DAVID.
